# Ouabain’s Influence on TRPV4 Channels of Epithelial Cells: An Exploration of TRPV4 Activity, Expression, and Signaling Pathways

**DOI:** 10.3390/ijms242316687

**Published:** 2023-11-24

**Authors:** Arturo Ponce, Isabel Larre, Lidia Jimenez, Maria Luisa Roldán, Liora Shoshani, Marcelino Cereijido

**Affiliations:** 1 Department of Physiology, Biophysics and Neurosciences, CINVESTAV-IPN, Mexico City 07360, Mexico; lidia.jimenez@cinvestav.mx (L.J.); luisa.roldan@cinvestav.mx (M.L.R.); shoshani@cinvestav.mx (L.S.); cereijido@cinvestav.mx (M.C.); 2 Department of Physiology, Faculty of Medicine, Universidad Nacional Autónoma de Mexico (UNAM), Mexico City 04510, Mexico; perezm@marshall.edu; 3 Department of Clinical and Translational Science, Joan C. Edwards School of Medicine, Marshall University, Huntington, WV 25755, USA

**Keywords:** ouabain, epithelial cells, TRPV4 channels, whole-cell patch clamp, ion currents, Na^+^/K^+^-ATPase

## Abstract

Ouabain, a substance originally obtained from plants, is now classified as a hormone because it is produced endogenously in certain animals, including humans. However, its precise effects on the body remain largely unknown. Previous studies have shown that ouabain can influence the phenotype of epithelial cells by affecting the expression of cell–cell molecular components and voltage-gated potassium channels. In this study, we conducted whole-cell clamp assays to determine whether ouabain affects the activity and/or expression of TRPV4 channels. Our findings indicate that ouabain has a statistically significant effect on the density of TRPV4 currents (dI_TRPV4_), with an EC50 of 1.89 nM. Regarding treatment duration, dI_TRPV4_ reaches its peak at around 1 h, followed by a subsequent decline and then a resurgence after 6 h, suggesting a short-term modulatory effect related to on TRPV4 channel activity and a long-term effect related to the promotion of synthesis of new TRPV4 channel units. The enhancement of dI_TRPV4_ induced by ouabain was significantly lower in cells seeded at low density than in cells in a confluent monolayer, indicating that the action of ouabain depends on intercellular contacts. Furthermore, the fact that U73122 and neomycin suppress the effect caused by ouabain in the short term suggests that the short-term induced enhancement of dI_TRPV4_ is due to the depletion of PIP2 stores. In contrast, the fact that the long-term effect is inhibited by PP2, wortmannin, PD, FR18, and IKK16 suggests that cSrc, PI3K, Erk1/2, and NF-kB are among the components included in the signaling pathways.

## 1. Introduction

Ouabain is an organic compound that has generated significant interest due to its diverse effects on various animal species, including humans. It can be toxic, have therapeutic potential, and cause physiological changes. As a member of the cardiac glycoside group, it was initially extracted from plants and later synthesized [1,2,3,4,5,6]. Although it was originally used as a medicinal aid to enhance heart muscle contraction, its use as medicine has declined because of its narrow therapeutic index [7]. Interestingly, it has been reported that some mammalian species, including humans, naturally produce ouabain, which is found in blood in picomolar to nanomolar concentrations. This has led to the consideration of it as a new steroid hormone, although its specific physiological functions are not yet well understood [8,9].

We have studied the influence of ouabain on epithelial physiology using MDCK, a cell line derived from a dog’s kidney [10,11,12], as our experimental model. Thus, we have shown that ouabain, at concentrations in the nanomolar range, influences the epithelial phenotype by modulating the expression of molecular components related to contacts and communication between cells. Specifically, we have described that ouabain (1) boosts intercellular connections facilitated by tight junctions, as proven by an increase in transepithelial electrical resistance (TER), a simultaneous decrease in the movement of dextran between cells, and an increase in the expression of claudins 1, 2, and 4 [13]; (2) induces alterations in adherens junctions, resulting in heightened expression of E-cadherin, β-catenin, and γ-catenin [14]; (3) fosters the enhancement of gap junctional communication among cells forming fully developed epithelial monolayers [15] by modifying the distribution of Cx32 and Cx43 within the membrane [16]; and (4) expedites the recovery of the epithelial phenotype in cells seeded at confluence after trypsinization, as indicated by the presence of an apical cilium in MDCK cells [17]. Apart from its involvement in regulating intercellular contact components, we have found that ouabain also influences the expression of voltage-gated potassium channels, entailing Na^+^/K^+^-ATPase acting as a receptor and the engagement of multiple signaling pathways, including cSrc, PI3K, Erk1/2, NF-κB, and β-catenin [18].

Based on this background, we have now tested the influence of ouabain on the expression of transient receptor potential vanilloid 4 (TRPV4), a type of ion channel which has been shown to be endogenously expressed in MDCK epithelial cells [19,20]. TRPV4 channels are expressed by various cells across the body, including the nervous system, skin, and vasculature. They react to various physical and chemical triggers, including temperature shifts, mechanical pressure, and osmolarity [21,22,23,24]. Despite being a type of nonselective cation channel, the activation of TRPV4 results in the entry of calcium ions, initiating various cellular reactions [25].

## 2. Results

### 2.1. MDCK Cells Cultured as Mature Epithelial Monolayers Exhibit Ion Currents Attributable to TRPV4 Channels

**Figure 1:**
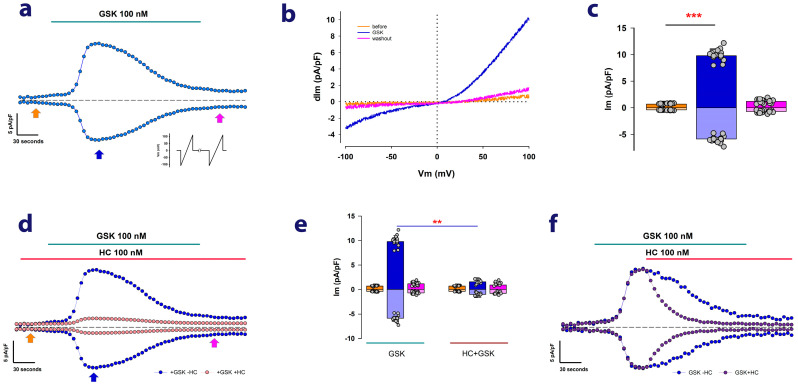
MDCK cells express ionic currents attributable to TRPV4 channels. (**a**) Representative example of continuous measurements of ion currents obtained by whole-cell patch clamp. The blue circles represent the values of ion current density obtained at +100 and −100 mV after ramp stimulation given repeatedly every 5 s, as illustrated in the inset. The top line indicates the time of addition of GSK to the bath medium. The arrows at the bottom show the times at which the ion current recordings shown in Figure 1b were obtained. The dotted line indicates the level of zero current. (**b**) Superposition of traces of current in response to ramp-voltage stimulation, recorded at the times indicated by arrows of the corresponding color in Figure 1a. (**c**) Bar graph comparing the average values of current density obtained before, during, and after the addition of GSK to the extracellular medium, as indicated by the arrows in Figure 1a. Measurements and comparisons shown are for values obtained at both +100 mV and −100 mV. (**d**) Superposition of continuous measurements of current density at +100 and −100 mV comparing a trial in which only GSK was added to the medium (blue-colored circles) with another in which HC-067047 (cantaloupe-colored circles) was added to the bathing media before GSK. (**e**) Bar graph comparing the average values of current density before, during, and after the addition of GSK to the bathing media alone (left) or after the previous addition of HC-067047 (right). Asterisks denote a statistically significant difference (** denotes *p* < 0.01; *** denotes *p* < 0.001 after *t* test) between the bars indicated by lines below the asterisk. (**f**) Superposition of continuous measurements of dIM at −100 and +100 mV comparing the effect of adding only GSK to the extracellular media (blue circles) with that of adding HC-067047 after GSK (purple circles).

Firstly, to ascertain the presence of endogenous TRPV4 channels in MDCK cells, we conducted electrophysiological, whole-cell clamp (WCC) assays under biophysical and pharmacological conditions that facilitate the recording of ion currents attributable to TRPV4 channels. As a well-established approach, we examined whether GSK1016790A (GSK), a specific TRPV4 agonist [26,27], could elicit the generation of ion currents in MDCK cells cultured as mature epithelial monolayers (MEM). Figure 1a shows a representative example of the results obtained from these experiments. After achieving the WCC configuration, the cells were stimulated repeatedly with voltage pulses in the form of a ramp that, starting from a holding value of 0 mV, changes to −120 mV and then varies steadily up to +120 mV for a millisecond before returning to the holding value of 0 mV. These pulses were applied every 5 s.

In Figure 1a, the blue circles represent the values of membrane current at +100 and −100 mV relative to the cell’s membrane area, which is referred to as the density of membrane current (dIm). As Figure 1a shows, perfusion with 100 nM GSK leads to a gradual increase in dIm at both +100 mV and −100 mV, peaking approximately 2 min after the addition of GSK. Subsequently, despite the continued presence of GSK in the extracellular medium, dIm gradually decreases.

Figure 1b depicts the current traces recorded in response to ramp-voltage stimulation before and after the addition of GSK, as marked by arrows in Figure 1a. As noted, they display inward and outward rectification, a characteristic of TRPV4 currents that have been described as depending on the extracellular concentration of calcium [25,28]. These results then indicate that such currents are due to the presence of TRPV4 channels in the plasma membrane of MDCK cells. Figure 1c shows a bar chart comparing the average values (±S.E.) of dIm at −100 mV and dIm at +100 mV before and after the addition of GSK.

To provide further evidence that these currents are caused by the presence and activity of TRPV4 channels, we conducted experiments to test the effect of HC-067047 (HC), a compound that has been demonstrated to specifically inhibit TRPV4 currents [29,30]. Figure 1d shows two sets of continuous recordings of dIm at −100 and +100 mV, which have been overlaid for comparison. In one of these sets (represented by cantaloupe-colored dots), HC was perfused before GSK, resulting in a significant reduction of the transient increase that is typically observed when GSK is administered alone (represented by blue-colored dots). As shown in Figure 1e, the addition of HC significantly decreases the transient enhancement of dIm induced by GSK alone, turning it statistically insignificant. Moreover, Figure 1f demonstrates that when HC is administered 2 min after the administration of GSK, the magnitude of dI_TRPV4_ experiences a quicker decline compared with GSK alone. These results altogether demonstrate that MDCK cells, cultured as MEM, express TRPV4 channels endogenously.

Subsequently, we chose to use the value of current density, measured only at +100 mV two minutes after the addition of GSK to the bathing media (hereafter referred to as dI_TRPV4_), as an indicator of TRPV4 channel expression.

### 2.2. Ouabain Enhances the dI_TRPV4_ of Cells Cultured as Mature Epithelial Monolayers

**Figure 2:**
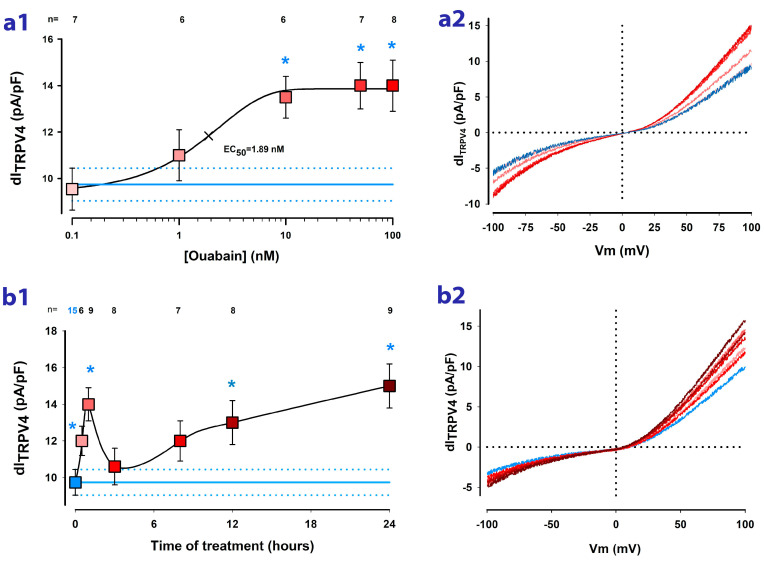
Ouabain affects the ion current density attributed to TRPV4 channels (dI_TRPV4_). The graph in (**a1**) plots the relationship between the average value of dIm at +100 mV and the concentration of ouabain added to the culturing media for 1 h before the trials. Measurements of dI_TRPV4_ were obtained from individual cells two minutes after the addition of GSK to the bathing media. Asterisks indicate a statistically significant difference between the average value of dI_TRPV4_ with and without ouabain treatment. The blue lines represent the average value (±S.E.) of dI_TRPV4_ obtained from cells not treated with ouabain. The numbers above each symbol indicate the number of trials. The graph in (**a2**) shows continuous traces of the current obtained in response to a ramp-shaped voltage stimulus for each of the ouabain concentrations depicted in (**a1**). The colors of the lines correspond to those of the symbols in (**a1**). The graph in (**b1**) describes the relationship between dI_TRPV4_ and the duration of treatment (in hours) with ouabain 10 nM. The asterisks indicate a statistically significant difference (*p* < 0.05) between the average value of dI at a given treatment time and that obtained from untreated cells whose value is shown by the blue box and the horizontal blue line. The graph in (**b2**) shows continuous traces of the current obtained in response to a ramp-shaped voltage stimulus for each of the treatment times depicted in (**b1**). The colors of the traces correspond to those of the symbols in (**b1**).

Next, to determine whether ouabain influences the activity of TRPV4 channels in MDCK cells constituted as mature monolayers, we conducted a comparative analysis of dI_TRPV4_ values between cells exposed to varying concentrations and durations of treatment with ouabain. On the one hand, we tested the effect of ouabain at various concentrations in the nanomolar range (0.1, 1, 10, 50, and 100 nM). As shown in Figure 2a1, treatment for one hour with ouabain induces an increase in dI_TRPV4_ that is statistically significant from 10 nM. An EC50 of 1.89 nM was calculated from fitting data to the sigmoidal Hill´s equation.

On the other hand, to know how the response varies depending on the time of treatment with ouabain, we conducted trials in which we measured dI_TRPV4_ in cells that had been incubated with 10 nM ouabain added to the culturing media for different times (hours) before the WCC trials. As illustrated in the graph of Figure 2b, from 30 min of treatment, there is a statistically significant increase that reaches a maximum at 1 h, followed by a decrease at 3 h of treatment; however, from 12 h, dI_TRPV4_ again increases significantly.

Therefore, these results indicate that ouabain effectively induces an enhancement in the activity of TRPV4 channels of MDCK cells cultured as mature monolayers. Moreover, the temporal response leads us to suppose that there are two types of modulations of TRPV4 channels, a short-term one that could be due to the modulation of activity or gating and a long-term one that could imply the modulation of expression of new channel units.

### 2.3. Ouabain Speeds up the Recovery of TRPV4 Channels Lost by Trypsinization

MDCK cells are known to undergo epithelial biogenesis when seeded in an appropriate substrate. They grow until they reach confluence and then form contacts with each other through the co-expression of various molecular components, such as tight junctions, adherens junctions, and gap junctions. The cells then polarize their membrane into an apical and basolateral domain to form a mature monolayer with epithelial properties (MEM) [31,32,33]. As part of the subculturing process, treatment with trypsin in the absence of extracellular calcium causes cells assembled as MEM to detach from each other and from the substrate and to lose their apical/basolateral polarization. However, once reseeded, these characteristics recover, and the cells once again conform to an MEM structure. Previous studies have shown that trypsinization also causes the decreased expression and altered localization of a variety of membrane components, including voltage-dependent potassium channels, and that to recover, cells require the synthesis and relocation of new channel units [34,35,36]. We have described evidence suggesting that ouabain can speed up the process of epithelial biogenesis. Firstly, ouabain can accelerate ciliogenesis, which is a sign of cell membrane apical/basolateral polarization after cells have contacted each other [17]; secondly, we have observed that ouabain can speed up the restitution of voltage-gated potassium channels that are lost during trypsinization [18].

Therefore, in this work, we aimed to investigate whether trypsinization affects the expression of TRPV4 channels, as estimated by dI_TRPV4_, and whether ouabain can influence the subsequent restitution process after reseeding cells. For this purpose, we measured dI_TRPV4_ before and at various time points (from 30 min to 24 h) after trypsinization in both untreated cells and cells treated with 10 nM ouabain. The ouabain was added to the culture medium at the time of reseeding and remained present until WCC trials.

**Figure 3:**
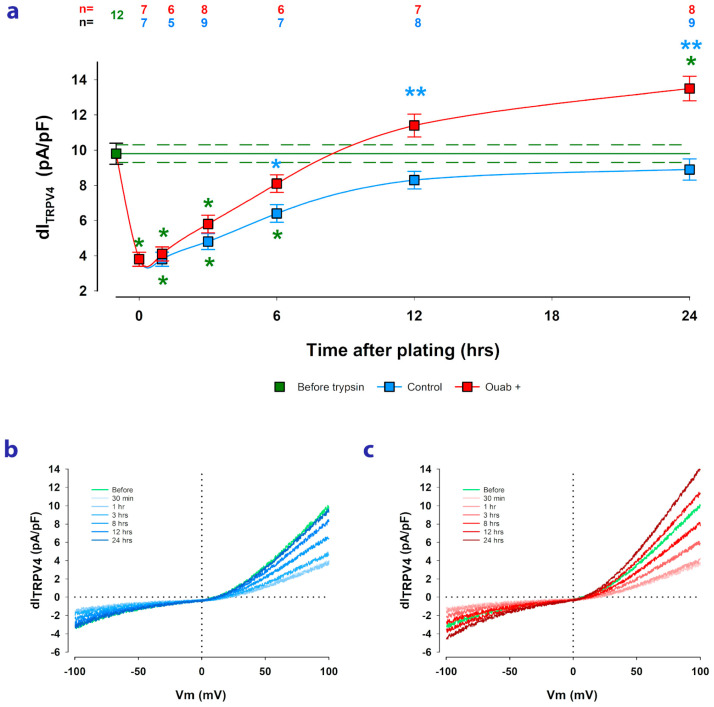
Ouabain accelerates the recovery of dI_TRPV4_ after the trypsinization and reseeding of MDCK cells. (**a**) Graph showing the relationship between the average value of dI and the time elapsed after reseeding, at confluence, of previously trypsinized cells. The blue squares represent the values obtained from cells not treated with ouabain 10 nM, while the red ones were obtained from cells to which ouabain was added from the moment of reseeding and remained present until the time of the recording. The green square and lines indicate the value obtained from cells in mature monolayers before trypsinization. Green asterisks denote a statistically significant difference (*p* < 0.05) between the value represented by the square and the corresponding value of the cells before trypsinization. Blue asterisks denote a statistically significant difference between the value of cells treated and not treated with ouabain at the same time after trypsinization. Numbers above the symbols indicate the number of repetitions of each experimental condition. (**b**) Superposition of representative traces of dI_TRPV4_ obtained at each of the different times after trypsinization from cells under control conditions. (**c**) Superposition of representative traces of dI_TRPV4_ obtained at each of the different times after trypsinization from cells treated with ouabain 10 nM.

As depicted in Figure 3a, the trypsinization process significantly reduced the average value of dI_TRPV4_ from 9.8 ± 0.6 pA/pF for cells in MEM before trypsinization to 3.8 ± 0.4 pA/pF for cells that had just been re-seeded. From then on, the dI_TRPV4_ recovered gradually in both ouabain-treated and untreated cells; however, cells treated with ouabain showed faster dI_TRPV4_ recovery than untreated cells, such that at 6 h after seeding, the mean value of dI_TRPV4_ obtained from ouabain-treated cells (8.3 ± 0.4 pA/pF) was significantly higher (*p* < 0.005) than the corresponding value from untreated cells (6.2 ± 0.4 pA/pF). These results suggest, therefore, that ouabain speeds up the recovery of dI_TRPV4_.

### 2.4. The Enhancing Effect of Ouabain on dI_TRPV4_ Relies on Contact between Cells

As previously described, when epithelial cells reach confluency, they contact each other and co-express cell–cell molecular structures. This results in the polarization of their membranes into an apical and a basolateral domain, forming an epithelial monolayer. A variety of membrane components require the cells to establish contact and polarize their membranes before they can be expressed [35,37,38,39,40].

To determine whether the expression of TRPV4 channels requires cell-to-cell contact, we conducted WCC trials to measure the dI_TRPV4_ from cells that had been seeded at low density (10% confluency) and that, therefore, had not reached the saturation necessary to contact each other. Also, since it is known that in the absence of extracellular calcium, cells do not establish intercellular contacts [41,42], we performed trials from cells that have been seeded at high density but kept deprived of extracellular calcium. For each of these conditions, we investigated how ouabain contributes to modifying the dI_TRPV4_ after 1 or 24 h of treatment.

**Figure 4:**
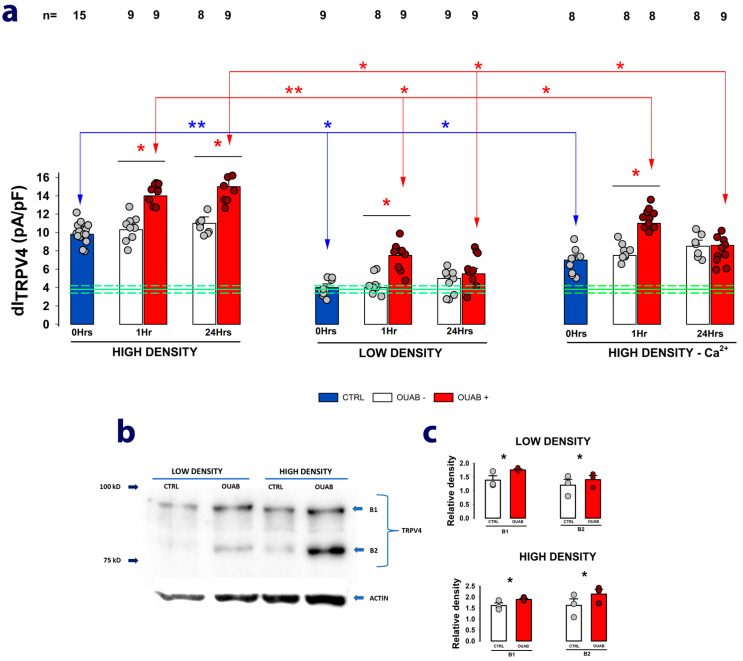
The effect of ouabain on dI_TRPV4_ depends on cell–cell contacts. (**a**) Bar graphs comparing the average (±S.E.) values of dI_TRPV4_ obtained from untreated (white bars) or treated (red bars) cells with ouabain 10 nM for 1 or 24 h. After trypsinization, the cells were seeded at high density (left), low density (middle), or high density but kept in a medium with low calcium (right). In all cases, the asterisks denote a statistically significant (*p* < 0.005) difference between the indicated group and its respective control, denoted by the blue bars. The numbers above the bars indicate the number of experimental repetitions. The dots denote the individual measurements. (**b**) Representative image of a Western blot assay comparing samples obtained from cells seeded at either low or high density and with or without ouabain. A pattern of two bands was obtained after staining with a TRPV4 antibody. (**c**) Bar graphs comparing the relative density of each of the two bands (B1 and B2) revealed by the anti-TRPV4 antibody in Western blot assays in both low-density (sparse) and high-density (De) seeded cells and treated for 24 h with ouabain 10 nM. The asterisks denote a statistically significant difference (*p* < 0.05) between the groups indicated by arrows. The dots indicate the individual values of each experimental repetition.

Figure 4a shows three sets of bars, which represent the average values of dI_TRPV4_ obtained from each experimental group treated or not with ouabain for 1 or 24 h. In each of them, the blue bars denote the mean values of dI_TRPV4_ obtained from the cells 12 h after being seeded but before adding ouabain 10 nM to the culture medium, while the following bars correspond to the values of dI_TRPV4_ without and with ouabain added to the culture medium and maintained for 1 or 24 h before the WCC assays.

A comparison of the blue bars shows that, after 12 h, cells seeded at low density did not significantly increase their dI_TRPV4_ (4.1 ± 0.4 pA/pF) compared with the value obtained just after trypsinization (3.8 ± 0.36 pA/pF) (*indicated by the green lines*). Cells seeded at high density but maintained in the absence of calcium were able to significantly restore their dI_TRPV4_ (6.5 ± 0.43 pA/pF), although to a lesser extent than those seeded at high density with calcium in the medium (8.5 ± 0.52 pA/pF). These results thus suggest that to restore the TRPV4 channels lost during the trypsinization process, cells require contact with each other.

The subsequent addition of ouabain to the culture medium caused a statistically significant increase in the average value of dI_TRPV4_ compared with untreated cells, but while in cells seeded at high density and kept in calcium-containing medium, this occurred both at 1 and 24 h, in cells seeded at low density or in cells seeded at high density but kept without calcium, ouabain induced an increase only at 1 h but not at 24 h. This then suggests that long-term treatment with ouabain stimulates the production of new ion channels but that this depends on whether the cells have already established contact with each other.

To strengthen the hypothesis that long-term treatment with ouabain promotes an increase in the expression of new TRPV4 channel units, we performed Western blot assays to quantify and compare the density of specific bands (revealed after probing with an anti-TRPV4 antibody) detected from lysate samples obtained either from cells assembled as a mature monolayer or from cells seeded at low density that therefore lacked cell–cell contacts. In both cases, cells were either treated or not with ouabain 10 nM for 24 h. As shown in the representative image in Figure 4b, a two-band pattern between 75 and 100 kD was obtained in all cases. In both cases, ouabain treatment induced a statistically significant increase in the density of both bands, as shown in the bar graphs in Figure 4c.

These results thus strengthen the hypothesis that long-term ouabain treatment increases TRPV4 expression.

### 2.5. Na^+^/K^+^-ATPase Acts as the Receptor for Ouabain-Mediated Modulation of TRPV4 Channel Activity and Expression

Next, to test whether Na^+^/K^+^-ATPase is the receptor through which ouabain exerts its modulatory effect on the expression of TRPV4 channels, we performed WCC assays to measure dI_TRPV4_ in a strain of MDCK cells (here called MDCK-R) that had been modified by altering the sequence of the alpha subunit of Na^+^/K^+^-ATPase, rendering it insensitive to the presence of ouabain [43,44]; therefore, it was expected that if Na^+^/K^+^-ATPase participates in the action of ouabain on dI_TRPV4_, this effect would not occur in MDCK-R cells compared with that observed in cells that do not have the same modification (referred to, for this comparison, as MDCK-W).

**Figure 5:**
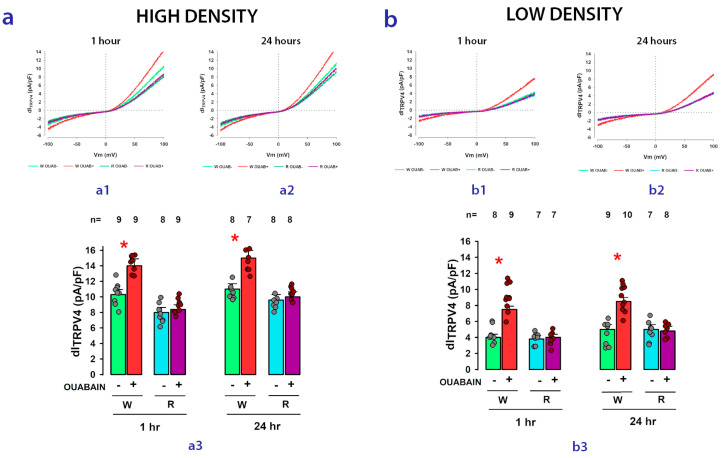
Na^+^/K^+^-ATPase acts as the receptor that transduces the modulatory effect of ouabain on the activity of TRPV4 channels. Sets **a** and **b** separately compare the effect of ouabain on dI_TRPV4_ in sensitive (W) and insensitive (R) cells seeded at high density (set **a**) or low density (set **b**). In both cases, the effect of ouabain was tested at 1 or 24 h of treatment. The top figures of each set show representative examples of ion currents recorded after ramp-shaped voltage stimulation at 1 (subsets **a1** and **b1**) or 24 h (subsets **a2** and **b2**) of treatment with ouabain. Bar graphs of each set (subsets **a3** and **b3**) compare the average values of dI_TRPV4_ under each of the experimental conditions described below the bars. The asterisks indicate a statistically significant difference (*p* < 0.05) of the group treated with ouabain compared with the untreated group. The numbers above the bars indicate the number of experimental repetitions of each group.

Figure 5 shows, in the form of bar graphs, the comparison of the results obtained for the average values of dI_TRPV4_ in MDCK-W (abbreviated W) and MDCK-R cells (abbreviated R) seeded either at high density (panel a) or at low density (panel b). In each case, the effect of ouabain on dI_TRPV4_ in the short (1 h) or long (24 h) term was statistically analyzed.

As represented here, while in MDCK-W cells, ouabain (10 nM) induces a statistically significant increase compared with its corresponding control, in MDCK-R cells, the same response is not observed under any conditions; neither at high nor low seeding density nor in the short or long term. These results support the hypothesis that Na^+^/K^+^-ATPase effectively participates as a receptor that initiates the signaling cascade through which the treatment with ouabain causes an increase in TRPV4 channel activity.

### 2.6. Signaling Pathways Involved in Short- and Long-Term Ouabain-Stimulated Enhancement of TRPV4 Activity

After verifying that ouabain influences the activity of TRPV4 channels and that Na^+^/K^+^-ATPase is the receptor, whose activation triggers this response, the next step was to find out which signaling pathways participate in this effect which, as we have described above, has two components: one in the short term, which is activated within 1 h, and a long-term one, which is gradually activated after at least 6 to 8 h of treatment with ouabain.

It is known that ouabain influences Na^+^/K^+^-ATPase activity in two ways. At doses in the micromolar range, it inhibits its functioning as a pump of Na and K ions, leading to an accumulation of intracellular calcium by overloading the sodium/calcium exchanger, thus inducing a positive inotropic effect, which accounts for its potentiating effect on cardiac cells. On the other hand, at concentrations in the picomolar to the nanomolar range, ouabain binding elicits the activation of Na^+^/K^+^-ATPase units located in caveolae/rafts to interact with neighboring lipid and membrane proteins (cSrc-kinase, PLC, and PI3K), leading to the assembly of signaling modules that, in turn, can activate a variety of signaling pathways, including the PI3K/Akt/mTOR and Src/Ras/Raf/Mek/Erk pathways [45,46,47].

To disclose which signaling pathways participate in the ouabain-induced enhancement of dI_TRPV4_, we conducted experiments with a set of chemical compounds that specifically inhibit various components of those pathways. Thus, we carried out WCC assays on cells in MEMs that were either treated or not with ouabain and/or each of these compounds, both in the short term (1 h) and long term (24 h).

**Figure 6:**
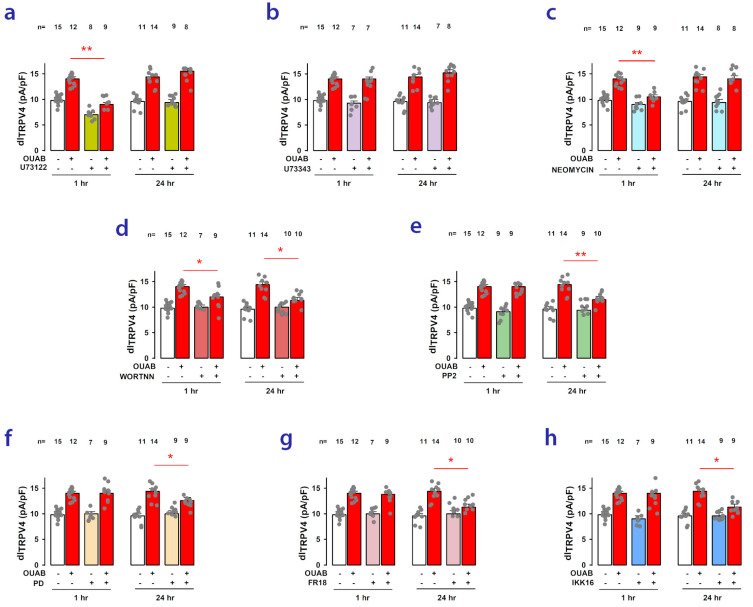
Involvement of signaling pathways in the modulatory effect of ouabain on dI_TRPV4_. (**a**–**h**) Bar graphs comparing the mean (±S.E.) value of dI_TRPV4_ in the presence or absence of different drugs inhibiting specific components of signaling pathways and/or in the presence or absence of ouabain, both at 1 and 24 h of treatment. In all graphs, the asterisks indicate that the presence of the given inhibitor produces a statistically significant reduction (*: *p* < 0.5 or **: *p* < 0.01) in the enhancement of dI_TRPV4_ induced by ouabain. The numbers above the bars represent the number of repetitions for each experimental group. Dots represent the individual dI_TRPV4_ values for each replicate in the experimental group.

Phospholipase C (PLC) is an enzyme responsible for facilitating the transformation of phosphatidylinositol 4,5-bisphosphate (PIP2) into inositol triphosphate (IP3) and diacylglycerol (DAG) [48,49]. As it has been described that PIP2 inhibits TRPV4 channel activity [50], we considered the possibility that the ouabain-induced enhancement of dI_TRPV4_ could be due to the depletion of PIP2 brought about by the activation of PLC. To test this, we conducted WCC trials to evaluate the effect of neomycin (500 μM) and U-73122 (50 μM), two different compounds that have been shown to inhibit PLC [51,52,53]. Additionally, we tested U-73343, a close structural analog of U-73122 that does not inhibit PLC, which is commonly used as a negative control [54]. As shown in Figure 6a–c, U-73122 and neomycin significantly reduced the increase in dI_TRPV4_ caused by ouabain in the short term but not in the long term, whereas the addition of U73343 did not produce a significant change. This supports the hypothesis that in the short term, the ouabain-induced enhancement of dI_TRPV4_ is due to the depletion of PIP2 caused by an increase in PLC activity.

This was further supported by the fact that wortmannin, a substance that irreversibly inhibits the activation of phosphatidylinositol 3-kinase enzymes (PIK3) [55,56] produced, in the short term, a result like that of PLC inhibitors, as shown in Figure 6d. PI3K is a kinase that transforms PIP2 into PIP3 [57,58], so its activation is also expected to partially deplete PIP2 reserves. Nonetheless, unlike PLC inhibitors, wortmannin also suppressed the ouabain-induced response in the long term, suggesting that the PI3K/Akt/mTOR pathway may be partially involved in the synthesis of novel TRPV4 channel units.

Next, we conducted tests to determine the involvement of cSrc by examining the effect of PP2, a powerful, reversible, ATP-competitive inhibitor of the Src family of protein tyrosine kinases [59,60]. As shown in Figure 6e, PP2 was able to inhibit the long-term response to ouabain but not the short-term response. A similar result to the one caused by PP2, i.e., suppression of the effect caused by ouabain in the long term but not in the short term, was obtained when the cells were treated with PD0325901 (PD) (Figure 6f) or with FR18024 (FR18) (Figure 6g), both potent inhibitors of MEK and ERK, respectively [61,62,63], suggesting the involvement of the RAS/RAF/MEK/ERK pathway.

Finally, we also tested the involvement of the nuclear-factor kappa-light-chain-enhancer of activated B cells (NF-κB), a protein complex that controls the transcription of DNA [64,65,66], by assaying the effect of IKK-16, a compound shown to inhibit IkB kinases (IKKs) [67,68]. As shown in Figure 6h, the fact that IKK-16 significantly suppressed the ouabain-induced response in the long-term favors the idea that the latter stimulates the synthesis of new TRPV4 channel units and that the NF-kB complex participates in this process.

## 3. Discussion

Cardiac glycosides, including well-known compounds such as ouabain, digoxin, and digitoxin, constitute a class of compounds derived from certain plants and are primarily utilized in the treatment of heart failure and specific arrhythmias [69,70]. Since the mid-20th century, it has been established that ouabain and similar cardiac glycosides disrupt the cellular ionic balance by inhibiting the pumping function of Na^+^/K^+^-ATPase. This inhibition results in increased myocardial contractility and a slower heart rate [69,70]. The subsequent discovery that Na^+^/K^+^-ATPase serves not only as a pump but also as a receptor, initiating diverse signaling pathways upon binding with ouabain or other cardiac glycosides, has led to the exploration of novel physiological, pathological, and potentially therapeutic functions associated with these compounds [71,72,73].

Recent revelations highlight that, beyond their well-known effects on the muscle tone of cardiac cells and smooth muscle, cardiac glycosides also influence significant cellular processes such as proliferation, survival, differentiation, and apoptosis. This has spurred interest in their potential as agents for anti-cancer treatment [74,75,76]. The impact of cardiac glycosides can vary based on their concentration, potentially leading to either harmful or beneficial outcomes. At micromolar levels, they hinder the pumping function of Na^+^/K^+^-ATPase, while at nanomolar doses, they prompt Na^+^/K^+^-ATPase to initiate various signaling pathways [77,78]. Some of these pathways involve the movement of ions, particularly sodium, potassium, and calcium, with the latter serving as a signaling messenger [79,80,81]. It is noteworthy that, among the various processes affected by ouabain, only a limited number of cases have documented its role in regulating ion-channel activity [18].

Addressing this matter, a recent investigation explored how ouabain influences the expression of voltage-gated potassium channels in epithelial cells. Using electrophysiological techniques, specifically whole-cell patch clamp, the study demonstrated that ouabain, at concentrations within the nanomolar range, induces alterations in the current density generated by these channels. However, the capacity of ouabain to modulate these channels is shown to depend, in part, on cellular connectivity and communication, particularly in the formation of an epithelial monolayer.

The focus of this study is on transient receptor potential vanilloid 4 (TRPV4) channels, a type of nonselective cation channel crucial in various physiological processes, including the sensation of mechanical and osmotic stimuli, as well as in the regulation of calcium signaling [21,22,23,82,83]. These channels are widely distributed in various cell types, including osteoblasts, chondrocytes, neurons, cardiac and muscle cells [84], as well as epithelial tissues in organs such as kidneys, lungs, spleen, skin, or sweat glands [85,86,87].

Previous studies have indicated that MDCK cells endogenously express TRPV4 channels [19,20]. However, their biophysical properties and the potential influence of ouabain on their expression or activity had not been investigated. Utilizing the whole cell clamp electrophysiological technique, this study demonstrated that mature epithelial monolayers of MDCK cells exhibit ion currents linked to the presence of TRPV4 channels, referred to as ITRPV4. Activation of these channels by GSK1016790A, a compound known for its specificity in activating TRPV4 channels [26,27], was confirmed. The study also established that ouabain indeed influences TRPV4 activity in a manner dependent on both concentration and time. The ion-current density attributable to TRPV4 channels (dI_TRPV4_) exhibits an increase when cells are exposed to ouabain concentrations ranging from 2 to 100 nM. This response is dependent on the duration of treatment, showing a kinetic profile with an initial peak at 1 h, followed by a decrease and subsequent increase after 6 h, extending up to 24 h. This kinetic profile suggests the presence of two types of modulation: a short-term modulation, possibly involving the channels already present in the membrane, and a long-term modulation, potentially requiring the synthesis of new channel units.

Given previous descriptions of ouabain stimulating the activation of PLC through NKA, leading to the transformation of PIP2 into IP3 and DAG [45,46,47,88,89], and the inhibitory effect of PIP2 on TRPV4 activity [50], this study evaluated the hypothesis that the short-term enhancement of dI_TRPV4_ is due to the depletion of PIP2 reserves. Inhibition of PLC by U73122 or neomycin suppressed this response, supporting the hypothesis. Additionally, treatment with wortmannin, an inhibitor of PI3K, a kinase that also utilizes PIP2 as a substrate, suppressed the short-term enhancement of dI_TRPV4_.

On the other hand, the hypothesis that the ouabain-induced long-term enhancement of dITRPV4 is due to the production of new channels, is based mainly on the observation of the sustained increase of dITRPV4 after 6 h, but also on the western blot results, which shows a statistically significant increase in the amount of protein recognizable by specific antibodies. Moreover, as we have seen, the results obtained with specific inhibitors of various components of signaling pathways, show that several of them inhibit the response in the long term but not in the short term, suggesting the participation of various signaling pathways that act to promote the synthesis of new components, including cSrc, given the result with PP2, MEK and Erk, given the results with PD and FR18. These results altogether suggest the involvement of the Src/Ras/Raf/Mek/Erk pathway. Also, the result that wortmannin inhibits the long-term response suggests the involvement of the PI3K/Akt/mTOR pathway. Interestingly, a somewhat similar result has been described in sensory neurons where ouabain modulates NaV1.8 sodium channels with a dual mechanism, a fast one involving modulation of gating properties and a delayed one involving control in channel expression [90].

This study also investigated how the trypsinization process affects TRPV4 channels and how cells recover their former characteristics as they grow and connect until they become a mature epithelial monolayer. The findings indicate a significant decrease in dI_TRPV4_ due to the trypsinization process, suggesting the loss of TRPV4 channels. However, these channels are gradually regained after several hours if the cells are seeded at high density or confluence. In this process of biogenesis, ouabain appears to play a role by accelerating the replacement of lost components, like voltage-gated potassium channels. The convergence of these findings implies that ouabain could potentially stimulate the reorganization of processes and constituents intrinsic to the epithelial phenotype, including ion channels such as voltage-gated potassium channels and TRPV4 channels. However, this does not necessarily indicate a functional correlation between the two types of channels, although exploring this possibility is worth considering.

MDCK cells, derived from the kidney, specifically from the distal convoluted tubule [91], which play a role in potassium reabsorption [92]. TRPV4 channels, along with TRPP2 channels, have been reported to be involved in this process [93,94]. Located in the apical membrane domain, TRPV4 channels sense elevated flows and respond by increasing intracellular calcium, which stimulates the high-affinity Ca2+-sensitive small-conductance channel KCa2.3 (SK3) [95,96]. The possibility exists that ouabain acts by modulating potassium secretion in the kidney, enhancing the response of TRPV4 to the mechanical stimuli produced by fluid flow in the tubule.

Another aim of this study was to compare TRPV4 expression between cells lacking cell contacts and those organized as an epithelial monolayer, as well as to investigate the potential influence of ouabain on these conditions. The results revealed that the magnitude of dITRPV4 was significantly lower in non-contact cells, whether they were seeded at sub-confluence or were seeded at confluence but deprived of external calcium. This finding suggests that cell-to-cell contact is crucial for the restoration of the cell phenotype and the re-establishment of lost channels during biogenesis. The data also indicated that ouabain prompted a significant short-term increase but not a long-term one, implying that the signaling mechanisms promoting the generation of new channels necessitate the establishment of intercellular contacts. Notably, while the whole-cell clamp trials did not exhibit a noteworthy rise in dITRPV4 following prolonged exposure to ouabain, the Western blot experiments did reveal a statistically significant increase in the amount of TRPV4 protein. This suggests that ouabain does indeed stimulate the expression of TRPV4, although these proteins may not be localized to the membrane. Reports indicating TRPV4 localization in the nucleus when cells are at sub-confluence suggest that these proteins may have regulatory functions in addition to their role as channels. Therefore, ouabain may stimulate the expression of TRPV4, either within the nucleus or in diverse cellular locations.

In summary, this study has demonstrated that MDCK cells express ion currents attributable to the presence of TRPV4 channels. Treatment with ouabain at nanomolar concentrations induces an increase in the magnitude of these currents, with a kinetic profile suggesting the activation of two forms of stimulation: a short-term modulation associated with the activity of channels already present in the membrane that is influenced by the availability of PIP2 and regulated by PLC, and a long-term modulation involving the synthesis of new TRPV4 channel units. The involvement of various signaling pathways, including RAS/RAF/MEK/ERK and PI3K/Akt/mTOR, adds complexity to the mechanisms underlying the modulation of TRPV4 channels by ouabain.

## 4. Materials and Methods

### 4.1. Cell Culture

In this study, we employed two types of MDCK cell strains. Most experiments were carried out using the MDCK-II strain, which is referred to as MDCK or MDCK-W and was obtained from the American Type Culture Collection. Additionally, we utilized an ouabain-insensitive strain, referred to as MDCK-R, kindly provided by Dr. Louvard of the Pasteur Institute. Both MDCK-W and MDCK-R cells were cultivated and maintained in a 5% CO_2_ environment at 36.5 °C using Dulbecco’s Modified Eagle Medium (DMEM. Cat. 430–1600, Life Technologies Inc Gibco/Brl Division, NY, USA) supplemented with penicillin–streptomycin (10,000 U/μg/mL, Cat. 15140122, Thermo Fisher Scientific, Waltham, MA, USA) and 10% fetal bovine serum (Cat. 26140079, Thermo Fisher Scientific, Waltham, MA, USA)).

For electrophysiological assessments, cells were seeded onto glass coverslips placed within 24-well culture plates either at high density (80% confluency) or low density (10% confluency).

### 4.2. Electrophysiological Recording of Cells

Using established protocols detailed elsewhere [18,35,97], ion currents were recorded employing the whole-cell patch clamp technique. Coverslips containing MDCK cells were positioned within a recording chamber mounted on the stage of an inverted microscope (Diaphot 300, Nikon, Japan). The chamber was filled with extracellular solution (see Solutions) and continuously perfused. For electrical contact between the bathing solution and the reference electrode, a U-shaped glass tubing containing 2% agarose in 500 mmol/L KCl was employed. The reference electrode was placed within a chamber containing KCl 500 mM. Micropipettes, made from borosilicate glass tubing (cat. 34500-99, Kimble Chase, Vineland, NJ, USA), were produced using a horizontal puller device (P-87, Sutter Instrument Co., Novato, CA, USA) to attain a tip resistance of 2–5 MΩ. These were then backfilled with intracellular solutions (see Solutions) and attached to a piezoelectric-driven micromanipulator (PCS6000, Burleigh Co., Bismarck, ND, USA) via a pipette holder. The pipettes were guided to the cells, and patch rupture was initiated by suction once the gigaseal exceeded values of 2 GΩ (typically 5 GΩ). The stimulation protocol consisted of a series of ramp pulses, which started from a holding potential (Vh) of 0 mV, switched to −120 mV, then rose steadily up to +120 mV at a rate of 4 mV/millisecond before switching back to 0 mV. These pulses were repeated every 5 s.

The voltage-pulse protocols and recording of ion currents were made with a patch-clamp amplifier (8900, DAGAN Corp, Minneapolis, MN, USA) controlled by a dedicated software suite (pclamp 8.0, Axon Instruments Inc., Burlingame, CA, USA).

### 4.3. Measurement of Membrane Area

Current capacitive transients, resulting from hyperpolarizing square-voltage pulses ranging from −100 to −110 mV, were generated and captured at a frequency of 10 KHz. Subsequently, the offline computation of membrane capacitance was conducted by integrating the capacitive transient’s area at the pulse’s onset. This integrated value was then divided by the pulse amplitude (−10 mV) using the following equation:

$$Cm=\frac{\int_{t0}^{\infty }Ic\cdot ∆t}{∆V}$$

where Cm denotes the membrane capacitance, Ic signifies the capacitive current, and ΔV represents the pulse amplitude (−10 mV). The integration calculation was performed using the Clampfit module of pClamp 8.0 (Molecular Devices). The cell membrane area was further calculated by assuming a specific capacitance of 1 µF/cm^2^ [98].

### 4.4. Western Blotting and Densitometry Analyses

Cells cultured on 35 mm petri dishes were rinsed three times with PBS and then subjected to cell lysis utilizing RIPA buffer (Santa Cruz Biotechnology, Santa Cruz, CA, Cat. sc-24948A) comprising a protease inhibitor mix for 15 min at 4 °C. The resulting cell lysate was collected into 1.5 mL microcentrifuge tubes, homogenized by passing through an insulin syringe 15 times, and subsequently centrifuged at 14,000× *g* rpm for 15 min at 4 °C. The supernatant was gathered, and the protein concentration was determined using the BCA Protein Assay (Thermo-Scientific Cat. 23228 and 23224). The samples were mixed with 4× loading buffer, heated for 5 min at 90 °C, and then loaded onto a 10% SDS-PAGE gel for subsequent Western blot analysis. The gel was transferred onto a PVDF membrane (Polivinil Difluor Amersham Life Science cat. RPN303F) using a semi-dry chamber (Biorad Trans-Blot SD Semi-Dry Electrophoretic Transfer Cell Cat. 1703940) at a constant 35 volts for 30 min. The PVDF membrane was then blocked with a solution of 5% semi-skimmed milk and 3% bovine serum albumin (BSA) (Bovine Serum Albumin Cohn fraction V powder, Equitech-Bio, Inc., Kerrville, TX, USA) for 30 min at room temperature. Subsequently, the primary antibodies, rabbit anti-TRPV4 diluted 1:3000 (Atomone Labs Cat. 853-871) and mouse anti-actin in antibody diluted 1:1000 (a kind gift of Dr. Manuel Hernández, CINVESTAV, Mexico), were incubated overnight at 4 °C, followed by ten washes with TBS-T (Tris-Buffered Saline Cat. sc-362305) with Tween 20 (Sigma Cat. P1379) at 0.01%. The secondary antibodies (Thermo Fisher Scientific, anti-rabbit HRP Cat. 656,120 and anti-mouse HRP Cat. 626520) were added and incubated for one hour at room temperature, followed by ten washes with TBS-T. Finally, the membranes were developed using Immobilon Western (Cat. WBKLS0500, MILLIPORE) and the immunoblots were imaged by FUSION FX6-XT and quantified by densitometry using Fiji 1.0 software.

### 4.5. Solutions

The pipette solution was composed of 20 mM CsCl, 100 mM cesium aspartate, 1 mM MgCl_2_, 10 mM Hepes, 4 mM Na2ATP, 10 mM 1,2-bis(2-aminophenoxy)ethane-N,N,N′,N′-tetraacetate (BAPTA), and 0.08 mM CaCl_2_, pH 7.4, adjusted with CsOH. The extracellular solution comprised 150 millimolar (mM) sodium chloride (NaCl), 6 mM cesium chloride (CsCl), 1 mM magnesium chloride (MgCl_2_), 5 mM calcium chloride (CaCl_2_), 10 mM glucose, and 10 mM Hepes, with a pH of 7.4 adjusted using NaOH. The osmolarity of this solution, determined using a vapor pressure Wescor 5500 osmometer (Schlag, Gladbach, Germany), was found to be 320 ± 5 milliosmolar.

### 4.6. Chemicals and Drugs

Sigma-Aldrich was the source of all salts, chemicals, and drugs utilized in this study. Catalog numbers, along with the stock concentration (in DMSO), and the administered dose are provided in Table 1.

### 4.7. Analysis of Data

The ionic current values were measured with the Clampfit module of pClamp 8.0 (Molecular Devices. Multiple comparison statistical tests (ANOVA) and paired tests (Student-t) were performed with EXCEL’s statistical analysis modules (Microsoft 365) and with Sigmaplot for Windows version 15.

## 5. Conclusions

The findings from this study’s data indicate the following: (1) MDCK cells demonstrate ionic currents due to the presence of TRPV4 channels. (2) Ouabain, when present in nanomolar concentrations, affects the expression and activity of these channels. (3) Ouabain’s effects occur both in the short and long term. In the short term, the modulation of TRPV4 activity by ouabain depends on the PLC-regulated availability of PIP2, which is activated by Na^+^/K^+^-ATPase. In the long term, this modulation involves the activation of signaling pathways like RAS/RAF/MEK/ERK and PI3K/Akt/mTOR, influencing the expression of new TRPV4 channel units. (4) Besides influencing TRPV4 channels’ activity and expression, ouabain also impacts the rate of recovery of lost channels during epithelial biogenesis after the trypsinization and subsequent reseeding of cells. (5) Ouabain’s modulatory capability is contingent on cell-to-cell contact.

## Figures and Tables

**Table 1:** List of chemical compounds used in this work.

Name	Cat. No.	Stock mg/mL	Dose
GSK1016790A	G0798	15	20 nM
HC-067047	SML0143	15	150 nM
U73122	U6756	0.5	2 µM
U73343	U6881	0.5	2 µM
Neomycin	1405-10-3	10	50 µM
Wortmannin	W1628	14	10 nM
PP2	P0042	1.4	100 nM
PD 098,059	P215	30	7 µM
FR180204	SML0320	25	400 nM
IKK-16	SML1138	10	200 nM

## Data Availability

Data are contained within the article.
